# Prediction of Delayed Neurodevelopment in Infants Using Brainstem Auditory Evoked Potentials and the Bayley II Scales

**DOI:** 10.3389/fped.2020.00485

**Published:** 2020-08-21

**Authors:** Xiaoyan Wang, Xianming Carroll, Hong Wang, Ping Zhang, Jonathan Nimal Selvaraj, Sandra Leeper-Woodford

**Affiliations:** ^1^Department of Child Health, Hubei Maternal and Child Health Hospital, Wuhan, China; ^2^Department of Public Health, Mercer University College of Health Professions, Atlanta, GA, United States; ^3^College of Life Science, Hubei University, Wuhan, China; ^4^Department of Biomedical Sciences, Mercer University School of Medicine, Macon, GA, United States

**Keywords:** pediatric neurodevelopment, brainstem auditory evoked potentials (BAEP), Bayley Scales of Infant Development, premature birth, perinatal conditions

## Abstract

**Background:** Brainstem auditory evoked potentials (BAEP) provide an objective analysis of central nervous system function and development in infants. This study proposed to examine the relationship between infant BAEP values at age 6 months, and their neurodevelopment at age 2 years assessed by the mental development indices (MDI), a form of Bayley Scales of Infant Development. We hypothesized that in infants with BAEP values outside normal range, there may be neurodevelopmental delays, as shown by their MDI scores.

**Methods:** An exploratory investigation was conducted using preterm (28–36 weeks gestation; 95 cases) and term infants (≥37 weeks gestation; 100 cases) who were born with specific perinatal conditions. BAEP values were recorded in these infants from 1 to 8 months of age, and compared with MDI scores in these infants at age 2 years. A multivariate linear regressions model was performed to test the associations between all variables and MDI scores. Stratified linear regression was used to test the interactions between gestational age and BAEP values with MDI scores. Significance was determined at a *p* < 0.05.

**Results:** We found that BAEP values were inversely associated with MDI scores in premature infants (β = −1.89; 95% confidence interval = −3.42 to −0.36), and that the effect of gestational age and BAEP values on the MDI scores is decreased by 1.89 points due to the interaction between these two variables. In premature babies, the lower the BAEP value below the mean, the greater the decrease in MDI score at age 2 years. Asphyxia and lower socioeconomic status in the family were also covariates associated with lower MDI scores at age 2 years.

**Conclusion:** The data provided evidence that BAEP values outside the normal range in premature infants at age 6 months may predict developmental delays in cognitive and motor skills, as shown by MDI scores. We propose that BAEP assessment may be utilized as a potential indicator for neurodevelopment, and suggest that early intellectual and public health interventions should be encouraged to enrich neurodevelopment in premature babies with BAEP values outside the normal range.

## Introduction

There has been a rapid increase in survival rates of premature infants in recent decades because of the advancement in neonatal intensive care. Certain unexpected medical issues occur in newborn infants which affect the long-term learning ability in children (1–4). If not addressed properly in the early stages of infant development, these medical issues in early infancy can have long-term effects on neurodevelopment (1–6).

Abnormality in the brainstem auditory evoked potentials (BAEP) is considered to be an early indicator for cognitive related brain issues in premature infants and those with perinatal issues (7). BAEP may be used to assess auditory function in infants and children, and is considered as a clinically useful method for evaluation of cognitive development (8). In a number of studies, BAEP values have been found to be outside the normal range in individuals with autism, intellectual and language retardation, and attention deficit/hyperactivity disorder (9, 10). Because of the widespread acceptance of BAEP as a clinical tool assessing early cognitive brain issues, we proposed to utilize BAEP in our investigation of neurodevelopment in infants with perinatal issues.

The Bayley Scales of Infant Development (BSID) is considered a clinically valid measurement for assessing infant developmental progress (11–15). In our study, we used the modified Bayley II scale which is used in Chinese hospitals to assess the neurodevelopment of premature infants between 1 and 42 months of age (11–15). The BSID-II includes the mental developmental index (MDI), which indicates adaptive behavior, language, and exploration activities, and the psychomotor developmental index (PDI) to assess gross and fine movements (11–16).

Because perinatal issues in newborns may lead to higher risks for negative neurological effects in these infants (1), we propose that assessments of infant BAEP values, in combination with gestational age and certain perinatal conditions, may provide potential predictors for later neurodevelopmental delays. Beyond prematurity and perinatal conditions at birth, there are multiple individual factors that likely influence early cognitive development, including socioeconomic conditions (17, 18) and parental education (19–23). Because of this, our current analyses will also include certain socioeconomic conditions as factors in the prediction of early childhood cognitive development.

Monitoring these predictors could possibly guide us on how to improve the neurological development of those children at high risk for delayed development. Our study investigates the predictive value of comparing BAEP indices at age 6 months to the MDI and PDI scores at age 2 years, in term and preterm infants born with certain perinatal conditions. We hypothesized that in infants with BAEP values outside the normal range, there may be developmental delays in cognitive and motor skills, as shown by the MDI and PDI scores in these babies. We propose that by using these assessments, we may be able to predict alterations in the neurodevelopment of infants exposed to compromising medical conditions at birth.

## Materials and Methods

### Study Design and Sample Enrollment

This study was designed as an explorative investigation. We conducted this retrospective study at Hubei Maternal and Child Health Hospital, Wuhan, Hubei Province, China. The study samples included preterm infants (95 cases; 28–36 weeks gestational age), and term infants (100 cases; ≥37 weeks gestational age) who were born between June 1st, 2014 to October 31st, 2015.

Our hospital has 30,000 births per year, and accounts for half of all newborns in Wuhan. The incidence of premature infants born in the Obstetrics Department of our hospital is 3.0–8.0%. Our Child Health Department works together with the Neonatal Department to plan long-term follow-up programs for all infants with high-risk factors. The Neonatal Department of our hospital also accepts premature babies transferred from the other hospitals in Hubei province, and these infants are transferred to our Child Health Department for follow-up after age 1-month. The infants randomly selected for this study were among our long-term follow-up infant population. A total of 230 infants selected for this study were to be followed up to at least 2 years of age, with the repeated BAEP examinations done regularly. However, due to various reasons, including parents relocating to other provinces, or not wanting to continue the repeated BAEP tests, 35 infants were lost to follow-up. As a result, only 195 infants remained to complete our study. The consent rate for participation was 85.7%.

Because there is information sharing between the Neonatal Department and our Child Health Department, we were able to access the essential medical, socioeconomic, and demographic information on file for the newborns selected for our study. All parents of newborns with perinatal conditions had filled out questionnaires prior to the infant follow-up programs, and we did not see these until after our random selection of infants for the current study. The questionnaires included socioeconomic and demographic information. Gender was the only demographic variable in our study, and maternal education, paternal education, maternal occupation, paternal occupation, and household income were considered as SES variables in this study. Parental education was assigned as being either compulsory education (<9 years, low level), high school education (9–12 years, middle level), or some college or advanced training (>12 years, high level) (24). Parental occupation was assigned as three levels: unemployment, manual labor, and professional. Household income was defined as <3,000 Yuan/month, 3,000–5,999 Yuan/month, and ≥6,000 Yuan/month; three levels, in which 1.00 USD was equal to 6.23 RMB (Yuan) in 2015. Higher levels of parental education, professional jobs, and income ≥6,000 Yuan/month were indicative of higher SES as the reference category.

The assessment forms also included whether the mothers had issues of hypertension, diabetes, or history of miscarriage, threatened abortion, intrauterine distress, or jaundice. These maternal conditions were found in the mothers of both preterm and term infants, and future studies may provide further information on the effects of these maternal issues on neurodevelopment in preterm and term infants. The infants included in this current study experienced perinatal issues such as infection, jaundice, asphyxia, respiratory failure, or intracranial hemorrhage around the time of delivery, and they were followed in our clinic up to the age of 2 years. For these perinatal conditions, the disease classification method currently used in our hospital is The International Classification of Diseases, an internationally unified disease classification method developed by the World Health Organization (WHO). The 10th revision of the “International Statistical Classification of Diseases and Related Health Problems” (25) is common worldwide, and is collectively referred to as ICD-10. Our hospital used the ICD-10 to classify disease conditions according to the etiology, pathology, clinical manifestations, and anatomical location of the disease, making this an organized method for disease coding in our health systems. In both the preterm and term groups in our study, all infants had been given the appropriate interventions for their medical conditions at birth. For infants with staged developmental delay, our doctors and nurses provide special training programs such as help for 3-month-old infants who are unable to raise their head, and, for 6-month-old infants who cannot sit without being supported, we offer training sessions to help these infants sit alone.

For this study, the selected infants had recovered from their neonatal treatment, were discharged from the hospital, and their vital signs were stable. Because these infants had been at high risk during the perinatal period, follow-up of these infants continued at our hospital until they were 2 years old. The criteria for exclusion from our study were babies born in our hospital with hearing impairments, cerebral palsy, severe cardiopulmonary disease, severe malformation or genetic metabolic diseases. Our study included regular follow-up examinations, monitoring infant growth and development level, feeding and exercise guidance, health education guidance for parents, and early rehabilitation training for infants with poor development level. Our main task was to promote normal growth and development in all infants. Because all families of infants in this study were given the same intervention guidance, including nutrition advice, infant muscle movement training, and early parent-child education information, the intervention measures were not taken into account for our study analyses.

This investigation was approved by the Ethics Committee of Hubei Maternal and Child Health Hospital. Informed consent of parents was obtained for all infants. We only tested infants with perinatal issues, and followed the “Ethical Review Measures for Biomedical Research Involving Human Beings.” The principle of this ethical review is to respect the voluntary will of the subjects and abide by the principles of benefit, non-harm and justice. For this reason, our study had no control group of healthy infants because this would not be beneficial for normal infants to undergo the numerous tests and assessments.

Because of these required constraints on testing healthy infants, the controls used for this study were those normal scale ranges built into each of the assessment tests utilized for this study. For BAEP assessment of hearing loss in infants, it is clinically acceptable to check the latencies of waves I, III, and V, interpeak latencies of I–III, III–V, and I–V for abnormalities of BAEPs (7, 8, 26–28). For this assessment, wave latencies of III and V, and interpeak latencies of more than two standard deviations between waves I–III, and I–V, or III–V are considered as abnormal (7, 8, 26–28). The BSID II used for neurodevelopment assessment includes the mental development index (MDI) indicating the scale of adaptive behaviors, language, and exploration activities, and the psychomotor development index (PDI) for assessment of gross and fine movements (11–16). The MDI and PDI scores assigned to each infant ranged from 120 (excellent development) to ≤ 69 (developmental delay) (11–16). All procedures and methods were performed in accordance with the approved guidelines.

### BAEP Assessment

**Figure 1:**
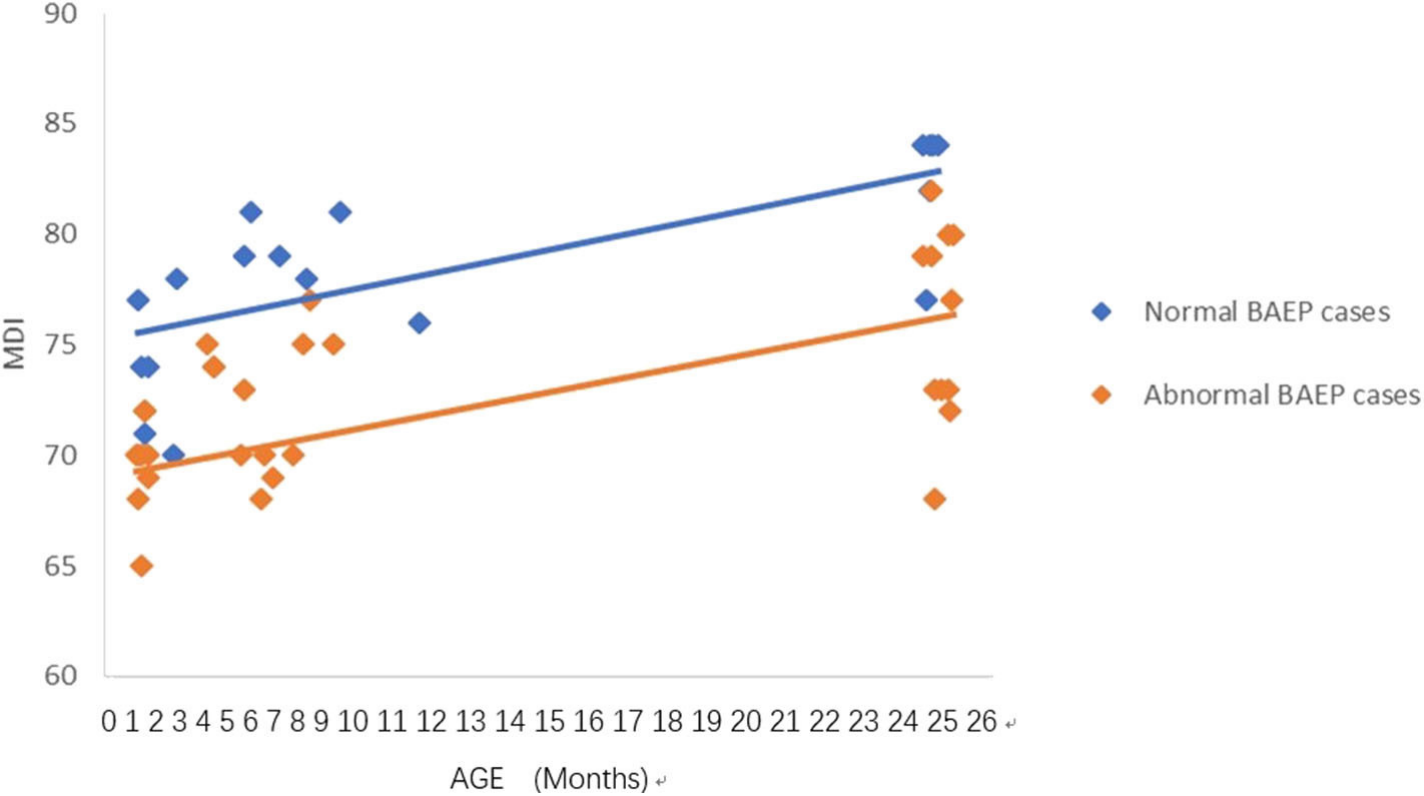
Mental Development Index (MDI) in preterm infants with perinatal asphyxia; comparisons of infants with Brainstem Auditory Evoked Potential (BAEP) within (normal), or outside (abnormal) normal range values. Each data point represents an individual infant case prior to 11 months of age and at 24 months. There is a trend of increased MDI in infants with perinatal asphyxia from below 1 year of age to age 2. From age one to two, there is a trend of higher MDI in infants with perinatal asphyxia and normal BAEP values compared with those infants with abnormal BAEP values.

**Figure 2:**
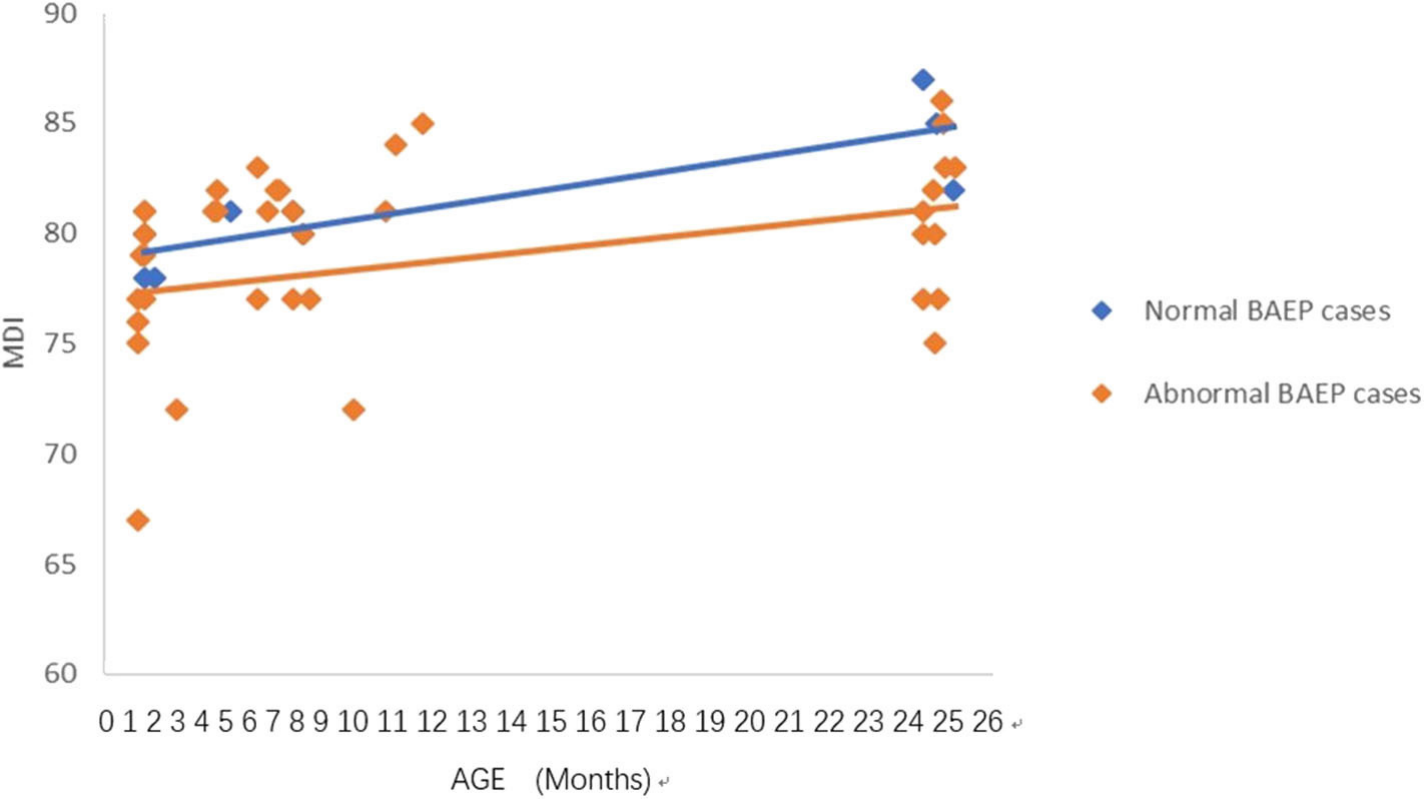
Mental Development Index (MDI) in preterm infants with perinatal respiratory failure; comparisons of infants with Brainstem Auditory Evoked Potential (BAEP) within (normal), or outside (abnormal) normal range values. Each data point represents an individual infant case prior to 11 months of age and at 24 months. There is a trend of increased MDI in infants with perinatal respiratory failure from below 1 year of age to age 2. From age one to two, there is a trend of higher MDI in infants with perinatal respiratory failure and normal BAEP values compared with those infants with abnormal BAEP values.

**Figure 3:**
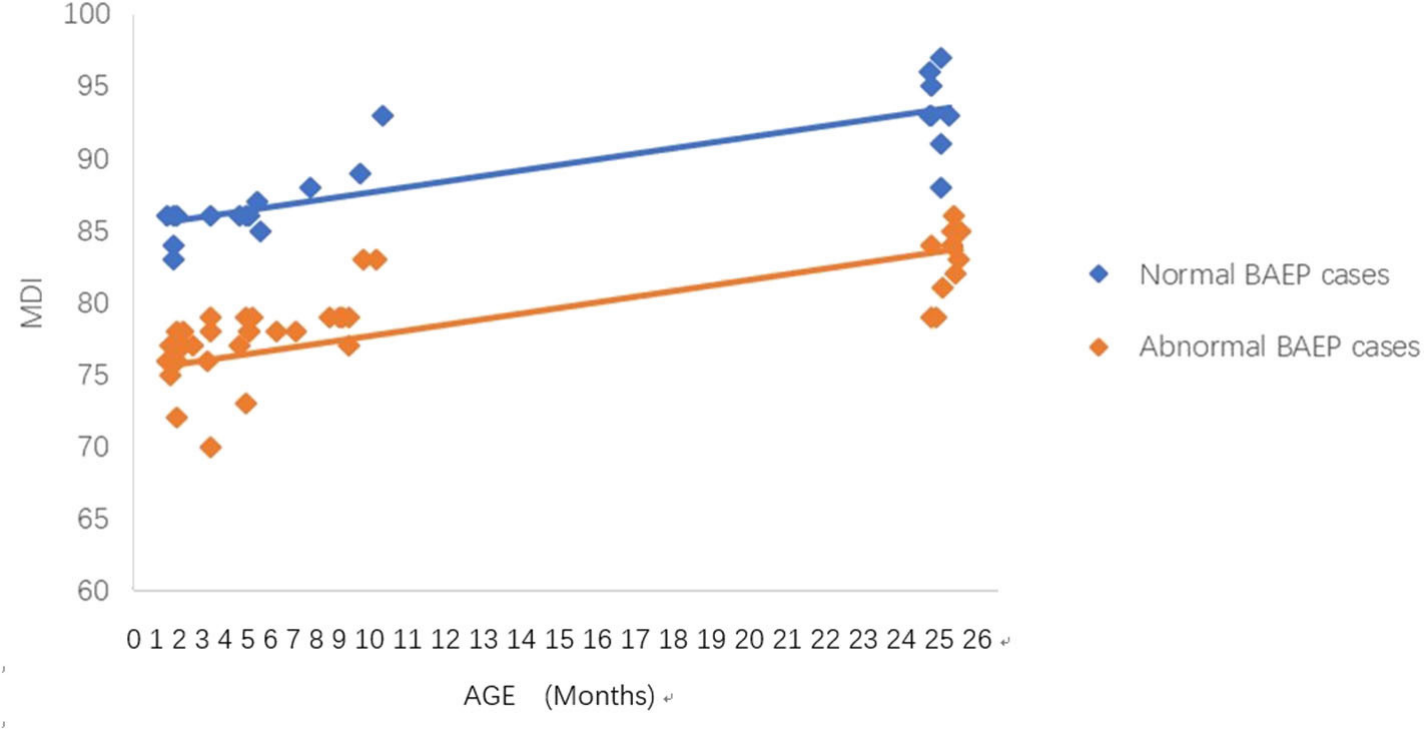
Mental Development Index (MDI) in term infants with perinatal asphyxia; comparisons of infants with Brainstem Auditory Evoked Potential (BAEP) within (normal), or outside (abnormal) normal range values. Each data point represents an individual infant case prior to 11 months of age and at 24 months. There is a trend of increased MDI in infants with perinatal asphyxia from below 1 year of age to age 2. From age one to two, there is a trend of higher MDI in infants with perinatal asphyxia and normal BAEP values compared with those infants with abnormal BAEP values.

**Figure 4:**
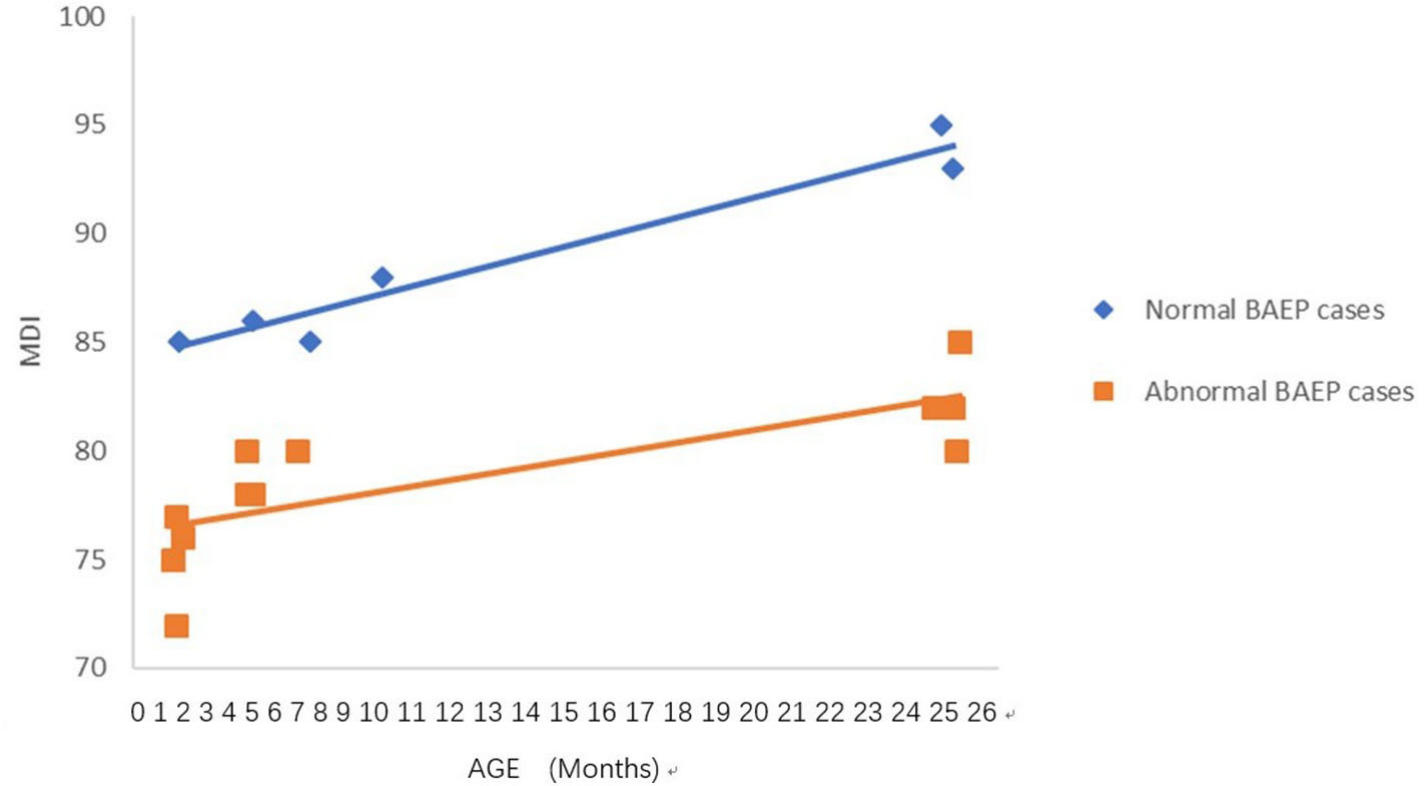
Mental Development Index (MDI) in term infants with perinatal respiratory failure; comparisons of infants with Brainstem Auditory Evoked Potential (BAEP) within (normal), or outside (abnormal) normal range values. Each data point represents an individual infant case prior to 11 months of age and at 24 months. There is a trend of increased MDI in infants with perinatal respiratory failure from below 1 year of age to age 2. From age one to two, there is a trend of higher MDI in infants with perinatal respiratory failure and normal BAEP values compared with those infants with abnormal BAEP values.

The auditory function of all infants in this study was assessed by using BAEP assessments at 1–8 months after birth. The BAEP assessment is considered an effective tool in screening for possible hearing loss in children with conditions such as meningitis, where it has been found that the frequency of BAEP impairment or hearing loss was 34.6 and 30.8%, respectively (29). The BAEP has been used to assess the hearing abilities in infants 6 months old, and in those older who have motor or intellectual problems (30). While BAEP assessments at 1, 3, 6, and 8 months are used to determine auditory function, age 6 months is the critical period for infant physical exams, and a number of deficits at birth are resolved by age 6 months. For these reasons, we focused our BAEP analyses in **Tables 5**–**7** and Figures 1–4 on the infants tested at age 6 months.

In our study, BAEP was recorded using the Navigator PRO brainstem auditory evoked potential system (Bio-logic Inc., USA). The recording electrode was placed in the midline of the forehead, and the reference electrode was placed at the bilateral mastoid. Electrode impedance was reduced to <5 kΩ, which was maintained during the whole session of the BAEP recording. The acoustic stimuli were given through TIP-50 earphones using a click sound stimulus. Band pass filter was 0.1–3 kHz, superimposition was 1,024–2,048 times, and stimulation rate was 30.1 times/s, and the sweep duration was 10 ms.

The BAEP assessments were performed in a sound-insulated room with a noise level below 30 dBA. The I, III, V wave latency and I–III, III–V, I–V wave interval were recorded under 80 dBnHL short-sound stimulation. The waves (I–V) are usually recorded in the first 10 ms following broad-band and high-intensity clicks. The latencies of waves I, III, and V, interpeak latencies of I-III, III–V, and I–V, and the amplitude ratio of wave V to wave I are assessed for abnormalities of BAEPs (7, 8, 26–28). Recordings are obtained and compared with respect to midline forehead and mastoids. Evaluating different components of the latencies and amplitudes of the waves allows for evidence of impaired neural function in the auditory brainstem as evidenced by delayed latencies and reduced amplitudes of the component waves (7, 8, 26–28).

BAEP impairment is determined by latencies of waves I, III, and V that are increased beyond 2 S.D. of age-matched normal infant values, with normal interpeak latencies of I–III, III–V, and I–V, and hearing thresholds elevated to above 40 dB. Wave latencies of III and V, and interpeak latencies of more than two standard deviations between waves I–III, and I–V, or III–V were considered as abnormal in our BAEP assessments (7, 8, 26–28). Abnormal BAEP is also noted by hearing thresholds increased to >40 dB, with normal latencies and interpeak latencies; or increased latencies of wave V or III (or both) and increased interpeak latencies of waves I-V and III-V, with elevated or normal hearing thresholds (7, 8). Duplicate recordings were made in response to each stimulus condition to recheck the reproducibility. BAEP was monitored in infants at 1, 3, 6, and 8 months of age.

### BSID II Neurodevelopmental Assessment

The Bayley Scales of Infant Development (BSID II) is used for neurodevelopment assessment, and is the most widely used measure to assess neurodevelopment of infants before the age of three (11–16). As noted in these previous studies, the BSID-I can be used for assessing infants between 2 and 30 months of age, while the BSID-II and Bayley-III can assess infants ranging between 1 and 42 months of age. In this study, we used BSID II for assessment of the infants, and analyzed the results with respect to different medical conditions of the infants during the time of delivery. The BSID-II is primarily used in China, and we used the revised edition prepared by Hunan Medical University (11–13). Our trained medical professionals are qualified to do the BSID-II assessment for neurodevelopment assessment in infants. The BSID II included the MDI, to evaluate and score adaptive behaviors, language and exploration activities, and the PDI to determine gross and fine movements (11–13). The MDI and PDI scores assigned are: ≥120 is excellent development, 119–90 is moderate development, 80–89 is between moderate and critical, 70–79 is critical, and ≤ 69 is developmental delay (11–14, 16).

### Data Analysis

The age of premature infants was calculated after correcting for gestational age. The BAEP assessments were presented from infants at 1–8 months of age, and the BSID II scores were from infants at ages 1 month, 3 months, 6 months, and 2 years. The relationship between the normal and abnormal BAEP assessment at the 6th month of age, and the MDI/PDI scores for neurodevelopment at age 2 years were also monitored with respect to the different conditions at birth. Because a recent meta-analysis study which reviewed BSID-II results indicated that MDI scores correlated strongly with later cognitive functions, explaining 37% of variance, while PDI scores only correlated later motor outcomes with 12% of the variance (16), we compared only the MDI scores with the BAEP assessments in **Table 5** and Figures 1–4. We did, however, analyze both MDI and PDI scores with the BAEP assessments in **Tables 6**, **7**. To eliminate evaluator bias in our study, only the researchers who designed the study and collected data knew the birth status of the infants, and the clinic doctors and nurses who were unaware of the perinatal status of the infants conducted the tests, or evaluated and recorded the assessment scores.

### Statistical Analysis

All statistical analyses were performed using SPSS version 19.0 (IBM SPSS Statistics, IBM Corporation, Armonk, NY). Chi-square tests and Cochrane's *Q*-tests were used for the comparisons of the same gestational groups over time. Using repeated measure ANOVA to analyze differences in the same groups over time, we examined MDI and PDI between term and preterm infants of different gestational ages (1 month, 3 months, 6 months, 8 months, and 2 years). Student *t*-tests were then used to examine the significance of the MDI at age 2 years, in the preterm and term infants exposed at birth to infection, jaundice, asphyxia, respiratory failure, or intracranial hemorrhage, with respect to whether each of the infants had normal or abnormal BAEP assessments at age 6 months. We constructed scatter plots to focus on the analyses of the MDI and BAEP assessments on preterm and term infants with asphyxia or respiratory failure because more infants with these conditions showed BAEP values outside of the normal range (see **Table 5**). We analyzed the trends in MDI scores at different ages in infants with perinatal asphyxia or respiratory failure, by comparing those with BAEP values outside the normal range (abnormal BAEP cases), vs. those with normal BAEP values (normal BAEP cases) (Figures 1–4).

Two steps of linear regression models were applied for further analyses of our data. In the first step, the association of all variables related to MDI and PDI was calculated in the multivariate linear regression model (31), including BAEP values at age 6 months and the other potential confounding factors, gestational age, SES (maternal education, paternal education, maternal occupation, paternal occupation, household income), and perinatal conditions (infection, jaundice, asphyxia, respiratory failure, intracranial hemorrhage). In the next step, we conducted the stratified linear regression model (32) to test our hypothesis that an interaction exists between gestational age and BAEP value at age 6 months on the MDI and PDI outcomes. We created the interaction term gestational age^*^BAEP for these two variables as the predictor, and MDI or PDI as the outcome in the stratified linear regression. Regression coefficients (β), 95% confidence intervals (CIs), and *p*-values were reported. All statistical tests were considered to be significant when less than an alpha level of 0.05 on a two-tailed test.

## Results

### Descriptive Statistics

#### Characteristics of Study Sample

Demographic information and SES variables of 95 preterm infants (28–36 weeks gestational age) and 100 term infants (≥37 weeks gestational age) are shown in Table 1. The percent within the different gestational groups was used for each demographic and SES variable to compare the gestational groups with respect to their socio-demographic conditions. There is a higher percent of less educated mothers (36.0%) and fathers (28.0%) in the very premature gestational group compared with the less educated mothers (5.0%) and fathers (8.0%) in the later stage gestational groups (Table 1). There is also a higher percent of low income families (40.0%) in the very premature gestational group compared with the low income families (10.0%) in the later stage gestational group (Table 1).

**Table 1:** Demographic information and socioeconomic status of 95 preterm infants and 100 term infants.

**Gestational age (*n*)**	**Gender**		**Socioeconomic status**									
	**Male *n* (%)**	**Female *n* (%)**	**Maternal education** ***n*** **(%)**		**Paternal education** ***n*** **(%)**		**Maternal occupation** ***n*** **(%)**		**Paternal occupation** ***n*** **(%)**		**Household income (Yuan/month)** ***n*** **(%)**	
28–32 weeks (25)	14 (56.0)	11 (44.0)	Low	9 (36.0)	Low	7 (28.0)	Unemployed	10 (40.0)	Unemployed	7 (28.0)	<3,000	10 (40.0)
			Middle	12 (48.0)	Middle	12 (48.0)	Manual labor	10 (40.0)	Manual labor	8 (32.0)	3,000–5,999	10 (40.0)
			High	4 (16.0)	High	6 (24.0)	Professional	5 (20.0)	Professional	10 (40.0)	≥6,000	5 (20.0)
33–34 weeks (28)	16 (57.1)	12 (42.9)	Low	0 (0)	Low	2 (7.1)	Unemployed	3 (10.7)	Unemployed	1 (3.6)	<3,000	1 (3.6)
			Middle	12 (42.9)	Middle	9 (32.1)	Manual labor	10 (35.7)	Manual labor	12 (42.8)	3,000–5,999	15 (53.6)
			High	16 (57.1)	High	17 (60.8)	Professional	15 (53.6)	Professional	15 (53.6)	≥6,000	12 (42.8)
35–36 weeks (42)	22 (52.4)	20 (47.6)	Low	2 (4.8)	Low	2 (4.8)	Unemployed	8 (19.0)	Unemployed	10 (23.8)	<3,000	2 (4.8)
			Middle	16 (38.1)	Middle	13 (31.0)	Manual labor	9 (21.4)	Manual labor	15 (35.7)	3,000–5,999	25 (59.5)
			High	24 (57.1)	High	27 (64.2)	Professional	25 (59.6)	Professional	17 (40.5)	≥6,000	15 (35.7)
≥37 weeks (100)	55 (55.0)	45 (45.0)	Low	5 (5.0)	Low	8 (8.0)	Unemployed	19 (19.0)	Unemployed	14 (14.0)	<3,000	10 (10.0)
			Middle	10 (10.0)	Middle	16 (16.0)	Manual labor	15 (15.0)	Manual labor	14 (14.0)	3,000–5,999	76 (76.0)
			High	85 (85.0)	High	76 (76.0)	Professional	66 (66.0)	Professional	72 (72.0)	≥6,000	14 (14.0)

*Term infants, ≥37 weeks gestational age; preterm infants, 28–36 weeks gestational age (number of weeks mother is pregnant). Maternal/Paternal education (number of years spent in school): Low ≤ 9 years; Middle 10–12 years; High >12 years. 1.00 USD equal to 6.23 RMB (Yuan) in 2015. n, number of infants in each category. %, percent within the different gestational groups for each socio-demographic characteristic*.

The clinical characteristics of 95 preterm infants and 100 term infants are shown in Table 2. Among the 95 preterm infants, the mean gestational age was 34.8 ± 3.82 weeks, the mean birth weight was 2.65 ± 0.41 kg, and the mean hospitalization time was 7.57 ± 2.35 days. The term and preterm infants had neonatal conditions including infection, jaundice, asphyxia, respiratory failure, and intracranial hemorrhage around the time of delivery. We calculated the percent within each gestational group instead of the total percent to make it easier to compare the frequency of the different perinatal conditions between the different gestational groups. When compared with the term newborns, the preterm infants in the study had more issues of jaundice (88.0%), followed by asphyxia (68.0 %), infection (60.0%), respiratory failure (52.0%), and intracranial hemorrhage (32.0%), as shown in Table 2.

**Table 2:** Clinical characteristics of 95 preterm infants and 100 term infants.

**Gestational age (*n*)**	**Delivery mode** ***n*** **(%)**		**Birth weight (kg)**	**Days of hospitalization**	**Infection**	**Jaundice**	**Asphyxia**	**Respiratory failure**	**Intracranial hemorrhage**
	**Vaginal delivery**	**Cesarean birth**			***n* (%)**	***n* (%)**	***n* (%)**	***n* (%)**	***n* (%)**
28–32 weeks (25)	8 (32.0)	17 (68.0)	1.97–2.42	7–22	15 (60.0)	22 (88.0)	17 (68.0)	13 (52.0)	8 (32.0)
33–34 weeks (28)	9 (32.1)	19 (67.9)	1.85–2.56	5–15	12 (42.9)	15 (53.6)	13 (46.4)	15 (53.6)	7 (25.0)
35–36 weeks (42)	13(31.0)	29 (69.0)	2.49–3.10	3–7	3 (7.1)	5 (11.9)	12 (28.6)	7 (16.7)	3 (7.1)
≥37 weeks (100)	18 (18.0)	82 (82.0)	2.90–4.10	1–7	3 (3.0)	10 (10.0)	26 (26.0)	7 (7.0)	2 (2.0)

*Term infants, ≥37 weeks gestational age; preterm infants, 28–36 weeks gestational age. n, number of infants in each category. %, percent within each gestational group to compare the frequency of the different perinatal conditions between the different gestational groups*.

#### BAEP Assessment

BAEP assessments were recorded in all term and preterm infants included in this study. Some infants in our study had to be excluded from the data analysis because they were unavailable for one or more of their BAEP assessment appointments. For this reason, 5% of these data were missing. We conducted Cochrane's *Q*-test for comparisons of the same gestational groups over time by analyzing the BAEP values outside the normal range across each of the infant age groups. We also conducted chi-square tests for comparisons of the BAEP values outside the normal range across the gestational age groups. When comparing these BAEP values in the different gestational age groups, significant results were observed at ages 3 months *p* = 0.001, 6 months *p* = 0.005, and 8 months *p* < 0.0001 (Table 3). Our results indicate that BAEP values outside the normal range tends to be higher and remains increased during the early growth period in preterm infants compared to term infants (Table 3).

**Table 3:** Brainstem Auditory Evoked Potential (BAEP) assessment for 95 preterm infants and 100 term infants.

**Gestational age (*n*)**	**BAEP value outside normal range at age 1 month *n* (%)**	**BAEP value outside normal range at age 3 months *n* (%)**	**BAEP value outside normal range at age 6 months *n* (%)**	**BAEP value outside normal range at age 8 months *n* (%)**	**Cochrane's *Q***	*p-value
28–32 weeks (25)	22 (85.7)	21 (82.7)	20 (80.0)	19 (75.0)	40.33	<0.001
33–34 weeks (28)	28 (100)	17 (61.8)	16 (57.0)	13 (45.0)	34.70	<0.001
35–36 weeks (42)	35 (82.6)	20 (47.4)	17 (42.0)	13 (32.0)	40.52	<0.001
≥37 weeks (100)	81 (80.7)	61 (60.5)	54 (54.1)	25 (24.5)	107.21	<0.001
χ^2^	2.16	7.62	6.55	8.79		
**p-value	0.080	0.001	0.005	<0.0001		

Comparisons of BAEP value outside normal range across the infant age groups are presented horizontally (Cochrane's Q-test, Cochrane's Q-value; *p-value). Comparisons of BAEP value outside normal range across the gestational age groups are presented vertically (chi-square-test, χ^2^-value; **p-value). Term infants, **≥**37 weeks gestational age; preterm infants, 28–36 weeks gestational age. n, number of infants in each category. %, percent within each gestational group. *p-value was obtained from Cochrane's Q-test; **p-value was obtained from chi-square test for categorical variables. Significant level p < 0.05.

#### Neurodevelopment Assessment Based on BSID-II

The BSID-II neurodevelopment assessment of the infants varied according to gestational age (Table 4). The MDI/PDI scores in the infants born at 28–32 weeks gestation showed an increased trend from the 1st month of age (78.36 ± 8.51/77.08 ± 8.89) to the 6th month (85.95 ± 5.73/85.33 ± 7.11), and this was increased further at age 2 years (90.66 ± 4.10/91.10 ± 3.26) (Table 4). In the infants born at 35–36 weeks, there were also trends for higher MDI/PDI scores during the growth period, at 1 month (81.80 ± 2.67/80.42 ± 2.64), 6 months (87.21 ± 1.11/86.24 ±1.15), and at age 2 years (94.13 ± 4.80/94.53 ± 4.72). In the term infants, MDI/PDI scores were higher than in preterm infants at age 1 month, and were increased to a greater extent at age 2 years (98.55 ± 4.64/99.81 ± 3.92). Overall, these data show that the neurodevelopment of premature infants of different gestational ages gradually increases with age, and that their developmental level gradually approaches that of the term infants (Table 4). However, when compared to the neurodevelopment of term infants at each of the respective age groups, these data also reveal that there are trends for differences in the neurodevelopmental levels of premature infants at 1-month, 3-months, 6-months, and 2 years of age (Table 4).

**Table 4:** Neurodevelopment assessment using Mental Development Index (MDI) and Psychomotor Development Index (PDI) for 95 preterm infants and 100 term infants.

**Gestational age (*n*)**	**MDI/PDI at age1 month(mean ±*SD*)**	**MDI/PDI at age3 months(mean ±*SD*)**	**MDI/PDI at age6 months(mean ± SD)**	**MDI/PDI at age2 years(mean ±*SD*)**	*F-value	*p-value
28–32 weeks (25)	78.36 ± 8.51/77.08 ± 8.89	82.60 ± 4.65/80.05 ± 5.31	85.95 ± 5.73/85.33 ± 7.11	90.76 ± 4.10/91.10 ± 3.26	29.02/21.86	<0.001/ <0.001
33–34 weeks (28)	80.98 ± 6.00/79.70 ± 5.58	85.76 ± 4.91/85.10 ± 4.10	86.57 ± 2.35/85.26 ± 2.60	90.93 ± 5.57/94.36 ± 6.67	38.12/49.29	<0.001/ <0.001
35–36 weeks (42)	81.80 ± 2.67/80.42 ± 2.64	84.96 ± 2.20/84.38 ± 2.44	87.21 ± 1.11/86.24 ± 1.15	94.13 ± 4.80/94.53 ± 4.72	51.53/90.42	<0.001/ <0.001
≥37 weeks (100)	90.22 ± 2.18/90.61 ± 3.26	91.11 ± 3.35/90.87 ± 2.59	90.35 ± 4.87/91.17 ± 3.37	98.55 ± 4.64/99.81 ± 3.92	27.70/99.65	<0.001/ <0.001
**F-value	73.19/93.97	101.96/47.57	47.96/101.47	18.10/27.87		
**p-value	<0.0001/ <0.0001	<0.0001/ <0.0001	<0.0001/ <0.0001	<0.0001/ <0.0001		

Comparisons of MDI/PDI across the infant age groups are presented horizontally (*F-value; *p-value). Comparisons of MDI/PDI across the gestational age groups are presented vertically (**F-value; **p-value). Term infants, **≥**37 weeks gestational age; preterm infants, 28–36 weeks gestational age. MDI or PDI Index: ≥120 is excellent, 119–90 is moderate development, 80–89 is between moderate and critical, 70–79 is critical, and ≤ 69 is developmental delay. n, number of infants in each category. p-value was obtained from ANOVA. Significant level p < 0.05.

#### Neurodevelopment MDI Scores With BAEP, and Prematurity, Asphyxia, and Respiratory Failure at Birth

In these comparisons, we used only the MDI scores because a recent study indicated that MDI scores correlated strongly with later cognitive functions, while PDI scores were less correlated with later motor functions (16). For this reason, we compared only the MDI scores with the BAEP assessment data in Table 5. As shown in this table, in the asphyxia and respiratory failure categories there were significant decreases in the MDI scores of both preterm and term infants with these perinatal conditions. We found that these conditions at birth may also impact the BAEP scores in both preterm and term infants (Table 5). In preterm infants who experienced asphyxia, the number of BAEP values outside the normal range at age 6 months (32 infants) was higher, and the MDI scores were significantly lower in this group at age 2 years (76.36 ± 3.66), when compared to those preterm infants with asphyxia at birth who were assessed with normal range BAEP values (10 infants; MDI scores, 82.30 ± 5.67) (Table 5). Similarly, in term infants with asphyxia at birth and BAEP values outside the normal range at age 6 months (13 infants), lower MDI scores were observed at age 2 years (83.12 ± 5.66), but significantly higher MDI scores were observed in the 13 infants with asphyxia at birth and BAEP in the normal range (93.42 ± 5.45) (Table 5). In the preterm infants who experienced respiratory failure at birth, the MDI score at age 2 years was 80.36 ± 7.66 in those with BAEP values outside the normal range at age 6 months (25 infants), but the MDI showed higher scores in those 10 preterm infants with respiratory failure at birth and normal range BAEP values (84.44 ± 5.55) (Table 5).

**Table 5:** Brainstem Auditory Evoked Potential (BAEP) value at age 6 months vs. Mental Development Index (MDI) at age 2 years in preterm infants and term infants with respect to perinatal condition.

**Gestational age (weeks)**	**Perinatal condition**	**BAEP value outside normal range at age 6 months *n* (%)**	**MDI at age 2 years (mean ± SD)**	**BAEP value normal range at age 6 months *n* (%)**	**MDI at age 2 years (mean ± SD)**
28–36	Infection	2 (1.0)	80.63 ± 5.21	28 (14.4)	89.75 ± 5.25
	Jaundice	3 (1.5)	83.43 ± 3.56	39 (20.0)	94.30 ± 7.74
	Asphyxia	32 (16.4)	76.36 ± 3.66^*^	10 (5.1)	82.30 ± 5.67^*^
	Respiratory failure	25 (12.8)	80.36 ± 7.66^*^	10 (5.1)	84.44 ± 5.55^*^
	Intracranial hemorrhage	2 (1.0)	90.35 ± 7.57	16 (8.2)	92.28 ± 4.52
≥37	Infection	1 (0.5)	90	2 (1.0)	93.89 ± 9.75
	Jaundice	1 (0.5)	92	9 (4.6)	93.62 ± 5.66
	Asphyxia	13 (6.7)	83.12 ± 5.66^*^	13 (6.7)	93.42 ± 5.45^*^
	Respiratory failure	5 (2.6)	81.15 ± 5.64^*^	2 (1.0)	95.78 ± 4.52^*^
	Intracranial hemorrhage	1 (0.5)	90	1 (0.5)	93

Comparisons of MDI scores of infants with BAEP value outside normal range vs. infants with normal range BAEP (*significant p-value). Term infants, ≥37 weeks gestational age; preterm infants, 28–36 weeks gestational age. MDI Index: ≥120 is excellent, 119–90 is moderate development, 80–89 is between moderate and critical, 70–79 is critical, and ≤ 69 is developmental delay. n, number of infants in each category. %, percent within each category.

* p-value was obtained from student t-test. Significant level *p < 0.05.

To analyze the trends in MDI scores at different ages, we constructed scatter plots to present data from the same infants prior to 11 months of age and at age 24 months (Figures 1–4). In these figures, with age as the horizontal axis and MDI as the vertical axis, the trend of MDI scores in specific infants as they increased in age was investigated in the different groups (term/preterm, respiratory failure/asphyxia, BAEP within normal range, or outside normal range). Figures 1–4 show that the MDI scores of infants with BAEP outside the normal range were lower than in infants with normal range BAEP in all four subgroups. Whether they are full-term or premature infants, and regardless of whether there is a history of asphyxia or respiratory failure, the MDI scores of infants with BAEP outside the normal range did not reach the developmental level of infants with normal range BAEP values (Figures 1–4).

### Multiple Linear Regression Analysis

#### Multivariate Linear Regression Model

Table 6 shows the results of two multivariate linear regression models in the overall sample of infants in our study, with all factors as the independent variables, and MDI and PDI as the outcome variables. The independent variables entered into the model included gestational age, SES (maternal education, paternal education, maternal occupation, paternal occupation, household income), perinatal conditions (infection, jaundice, asphyxia, respiratory failure, intracranial hemorrhage), and BAEP values at 6 months of age. Model 1 entered the main effects of all factors related to MDI scores. Model 2 entered the main effects of all factors related to PDI scores.

**Table 6:** Multivariate linear regression model.

**Factors**	**MDI (*****n*** **=195) Model 1**					**PDI (*****n*** **=** **195) Model 2**				
	**Unstandardized coefficients**		**95% CI**	***t***	***p***	**Unstandardized coefficients**		**95% CI**	***t***	***p***
	**β**	**SE (b)**				**β**	**SE (b)**			
Gestational age	0.36***	0.09	0.13 to 0.50	4.21	**0.000**	0.29***	0.08	0.07 to 0.41	3.70	**0.000**
Maternal education	1.50*	0.74	0.59 to 3.91	2.02	**0.045**	0.57	0.68	−0.91 to 2.14	0.84	0.403
Paternal education	−0.81	0.98	−2.45 to 1.54	−0.82	0.411	−0.40	0.90	−2.10 to 1.56	−0.44	0.660
Maternal occupation	−0.03	0.80	−2.57 to 0.94	−0.04	0.968	0.94	0.73	−0.76 to 2.47	1.29	0.199
Paternal occupation	1.76	1.05	−0.88 to 3.42	1.67	0.096	0.76	0.96	−1.29 to 2.65	0.79	0.430
Household income	1.42*	0.63	0.19 to 2.60	2.26	**0.025**	0.75	0.58	−0.63 to 1.94	1.30	0.195
Infection	1.42	1.04	−0.61 to 3.59	1.36	0.175	1.75	0.96	−0.16 to 3.69	1.84	0.068
Jaundice	0.41	0.78	−1.31 to 2.18	0.52	0.601	0.39	0.71	−2.05 to 1.16	0.55	0.586
Asphyxia	−1.27*	0.64	−2.45 to −0.27	−1.99	**0.048**	−0.77	0.58	−2.31 to 0.18	−1.32	0.189
Respiratory failure	−1.41	0.91	−3.24 to 0.43	−1.55	0.123	−0.56	0.83	−2.24 to 1.14	−0.67	0.501
Intracranial hemorrhage	−3.80	2.47	−8.99 to 0.95	−1.54	0.126	−1.56	2.26	−6.06 to 3.07	−0.69	0.492
BAEP (6 months)	−1.00	0.58	−1.96 to 0.36	−1.74	0.084	−0.86	0.53	−1.81 to 0.32	−1.63	0.106

All factors associated with Mental Development Index (MDI) and Psychomotor Development Index (PDI) at age 2 years. Gestational age—number of weeks mother is pregnant. BAEP (6 months)—Brainstem Auditory Evoked Potential at age 6 months. Model 1: Outcome—MDI; Model 2: Outcome—PDI. β–Unstandardized regression coefficient. SE(b)—Standard Error of value of b. Significant level *p < 0.05; ***p < 0.001 (bolded figures when p < 0.05).

In Table 6, we found that gestational age was very strongly associated with MDI scores (β = 0.36; 95% CI = 0.13–0.50) and PDI scores (β = 0.29; 95% CI = 0.07–0.41). We also found that SES indicators (maternal education and household income) are associated with MDI scores but not PDI scores, with respect to maternal education (β = 1.50; 95% CI = 0.59–3.91) and household income (β = 1.42; 95% CI = 0.19–2.60), respectively. In addition, our data showed that asphyxia was inversely associated with MDI scores (β = −1.27; 95% CI = −2.45 to −0.27), which means that infants born with asphyxia are predicted to have low MDI scores an average of 1.27 times more than the infants without asphyxia (Table 6). In the multivariate linear regression model, we did not find that the BAEP value at age 6 months was significantly related to MDI scores, but the *p* = 0.084 may possibly be due to the interactive correlations of two independent variables such as gestational age and BAEP value. In this case, we will continue to conduct further analyses to reveal the interactions between gestational age and BAEP values on MDI and PDI scores.

#### Stratified Linear Regression Model

Table 7 shows a summary of the results for the stratified linear regression models. In Model 3 and Model 4, gestational age^*^BAEP value at age 6 months was the predictive variable, and MDI and PDI were the outcome variables. The interactions of BAEP values and gestational age were estimated in MDI scores and PDI scores, respectively.

**Table 7:** Stratified linear regression model.

**Gestational age (*n*)**	**MDI Model 3**					**PDI Model 4**				
	**β**	***SE***	**95% CI**	***t***	***p***	**β**	***SE***	**95% CI**	***t***	***p***
28–36 weeks (95)	−1.89*	0.76	−3.42 to −0.36	−2.50	**0.015**	−0.74	0.68	−2.11 to 0.63	−1.13	0.263
≥37 weeks (100)	0.28	0.91	−1.52 to 2.08	−0.35	0.725	−0.77	0.85	−2.52 to 0.98	−1.23	0.223

Brainstem Auditory Evoked Potential (BAEP) at age 6 months in Preterm and Term Infants Associated with Mental Development Index (MDI) and Psychomotor Development Index (PDI) at age 2 years. Model 3: Outcome—MDI; Model 4: Outcome—PDI. Predictor variable—Brainstem Auditory Evoked Potential (BAEP) at age 6 months. Covariates entered into the model—SES (maternal education, paternal education, maternal occupation, paternal occupation, household income) and perinatal conditions (infection, jaundice, asphyxia, respiratory failure, intracranial hemorrhage). Term infants, ≥37 weeks gestational age; preterm infants, 28–36 weeks gestational age. n, number of infants in each category. β–Regression coefficient. SE—Standard Error. Significant level *p < 0.05 (bolded figures when p < 0.05).

We found that in the preterm infant group, the BAEP value at age 6 months was an independent factor and inversely associated with MDI scores at age 2 (β = −1.89; 95% CI = −3.42 to −0.36, *p* = 0.015; Table 7). In the term infant group, there was no statistical significance (β = 0.28; 95% CI = −1.52 to 2.08, *p* = 0.725) between BAEP value and MDI scores as shown in Table 7. These findings suggest that in premature infants there is a significant difference in the magnitude of the association between BAEP values at age 6 months and MDI scores in gestational age, and that a BAEP value outside the normal range at age 6 months may possibly be a predictor of lower MDI scores at age 2 years in these babies. As noted in Table 7, the effect of gestational age and BAEP on the MDI score is decreased by 1.89 points due to the interaction between these two variables, in that, the decrease in MDI score in infants with a lower BAEP value is dependent on gestational age, and vice versa. Also, the lower the BAEP value was below the mean, correlated with a greater decrease in MDI score with gestational age. There was no significant difference in the relationship between BAEP at age 6 months and PDI score in either preterm or term infants (Table 7).

## Discussion

In this study, we found that BAEP values at age 6 months in premature infants is inversely associated with MDI scores at age 2 years. The effect of gestational age and BAEP value on the MDI score is decreased by 1.89 points due to the interaction between these two variables (Table 7). In premature babies, the lower their BAEP values were below the mean, the more the decrease was in their MDI scores at age 2 years. These results support our hypothesis that BAEP values outside the normal range at age 6 months have a predictive effect on neurodevelopmental delay, as shown by MDI scores, especially in premature newborns. In this study, we found that prematurity in newborns was strongly associated with low MDI and PDI scores in these infants at age 2 years. We also found that infants who experienced asphyxia at birth were more likely to have low MDI scores at age 2 years. In addition, our data revealed that infants born in families from lower SES, as indicated by maternal education and household income, were more likely to have low MDI scores at age 2 years (Table 6).

### BAEP Value Predicts MDI Score in Premature Infants

There is increasing evidence that the issues related to cognitive and motor function performance in early childhood are related to clinical conditions during preterm or term birth (1, 33). Gestational age was found to have an effect on BAEP assessments in infants, and was also strongly associated with MDI and PDI scores as shown in our data (*p* = 0.000; Table 6). In our stratified multiple linear regression model, our data revealed that BAEP values outside the normal range in infants at age 6 months were more likely associated with developmental delays in cognitive skills, as shown by MDI scores in these infants (Table 7). In these analyses, we found that the BAEP values were inversely associated with MDI scores in preterm infants, but no significant differences were observed between BAEP and MDI scores in term infants. These data suggest that BAEP values at 6 months of age may be used as predictors for neurodevelopmental delay at age 2 years, especially for premature infants. We will expand the sample size in future studies to investigate whether this same significance is also present in term infants.

The younger the fetus, the more chances of brain cell damage caused by various conditions due to immature development (7, 34). Infants in these previous studies exhibited lower auditory function scores in the first year of infancy, and this was correlated with their lower MDI/PDI indicators of cognitive and motor development. Our current investigation provides evidence that infants may have issues of cognitive and motor function performance if they experience prematurity. A significant number of preterm infants in our study exhibited BAEP values outside the normal range in the first year of infancy, and this was correlated with their lower MDI/PDI scores in the BSID assessments of cognitive and motor development. We also observed that the majority of preterm infants who experienced asphyxia at birth and had BAEP values outside the normal range at age 6 months exhibited lower MDI/PDI scores, suggesting that there may be altered cognitive and motor functions, especially in language development in these infants at age 2. Our results indicate that BAEP values outside the normal range could be a potential indicator for altered cognitive and motor function in these infants at later ages.

In our study, we used the modified Bayley II scale which is used in Chinese hospitals to assess the neurodevelopment in premature infants. With our results utilizing the Bayley-II scale, we provided evidence that this assessment may be used to predict abnormalities in infant neurodevelopment, yet further studies are needed to compare with assessments reported using the Bayley III scale. Previous studies have shown that the Bayley-III scale could be effective in measuring developmental functions with respect to examiner observations, and parent-reported behaviors (12, 35). But, while this scale may be used to collectively assess language development, many larger studies are needed to prove its effectiveness (12, 35). Previous publications have indicated that the BSID-II has higher predictive abilities for future functioning, while others favor BSID-III (12, 13, 36, 37). There are indications that BSID-II might underestimate development and BSID-III might overestimate development (12, 13, 38). In previous studies, the Bayley III scale identified significantly fewer children with disabilities with respect to low birth weight preterm infants, and the authors proposed that intervention may be essential for these infants at the time of discharge from the neonatal intensive care unit (39, 40). The Bayley-II and brain magnetic resonance imaging to assess the neurodevelopmental outcomes have mostly been applied to infants exposed to asphyxia (41, 42). In our study using the Bayley-II assessment, we identified a number of cases with neurodevelopmental issues especially in the infants exposed to asphyxia.

The MDI/PDI scores of cognitive and motor function vary during the stages of early infant development (43, 44). A previous study indicated that variation in the PDI score for psychomotor function is less significant compared to the MDI score for mental/cognitive function in low birth weight infants (44). In this study, we found similar results consistent with this previous study that PDI score for psychomotor function is less significant compared to the MDI score (Table 7). In our current study, we found that preterm infants with BAEP values outside the normal range had significantly lower MDI scores, compared to those of term infants.

Studies using the Bayley II scale consistently identified high rates of cognitive impairments among preterm or low birth rate infants (45). In our study, the cognitive impairments tend be higher in preterm infants subjected to asphyxia or respiratory failure, based on the Bayley II assessment. Other studies have shown that there is a reduction in cognitive impairment level from 39% at 20 months to 16% at 8 years, based on the Bayley II assessment in extremely low birth weight infants (46). In our study, the MDI/PDI values for cognitive and motor scores that were observed at age 2 years might have shown increases at the later stages of development, if these infants had been exposed to early interventions such as cognitive and motor exercises, physical therapy sessions, and learning games with parents to improve their developing cognitive and motor skills.

### Asphyxia Associated With MDI Score

Our investigation has shown that perinatal issues such as prematurity and asphyxia may be negatively associated with MDI scores. Infants born with asphyxia had low MDI scores an average of 1.27 times more than the infants without asphyxia (Table 6). Previous publications have reported that as a result of perinatal asphyxia in infants, alterations of the putamen and thalamus of the brain, and atrophic areas in the brainstem were observed (47). Studies using magnetic resonance imaging in newborns with perinatal asphyxia showed lesions in basal ganglia, thalamus, brainstem tectum, parasagittal cortex, and the midline cerebrum, but found no lesions in basal ganglia and parasagittal regions (48). According to previous studies, perinatal asphyxia may cause lesions in the central generators of brainstem auditory-evoked response components, such as the cochlear nuclei, superior olive, and inferior colliculus (48). In addition, it was found that auditory-evoked response abnormalities in the brainstem occurred more frequently after severe, prolonged asphyxia (48).

Other studies have also proposed that following perinatal hypoxemia, damage to the neonatal auditory system, including the cochlea, may result in hearing deficits (48). Children who experienced perinatal asphyxia tended to exhibit hearing loss and neurodevelopmental deficits when compared to those not exposed to asphyxia (26, 34). In reported cases of perinatal asphyxia, infants generally recovered without neurological defects, but neurodevelopmental deficits due to brain hypoxia-ischemia were noted in some of these infants (27, 28).

In our current study, the data revealed abnormal brainstem auditory-evoked responses in infants exposed to perinatal conditions such as asphyxia, and we proposed that this auditory assessment may be used to predict delays in their neurodevelopment. Our results support using the early BAEP assessments in infants as indicators of possible delays in neurodevelopment of infants with perinatal conditions such as asphyxia, as well as, prematurity and respiratory failure. To further support this proposal, Figures 1–4 in our results show that the cognitive development of infants was lower in the infants who had BAEP values outside of the normal range, and that this was observed in all of the perinatal categories, including term, preterm, asphyxia, and respiratory failure. Further studies using a larger number of infants, including those with perinatal infection, intracranial bleeding, jaundice, and perhaps other conditions at birth will be pursued in future investigations.

### SES Related to MDI Scores

We had proposed that SES indicators are related to prematurity in newborns, and our data revealed that a higher percent of less educated mothers and low income families had infants in the very premature gestational group, when compared with less educated mothers and low income families with infants in the later stage gestational group (Table 1). After analyzing the data, we also found that SES indicators such as maternal education and household income were associated with infants having lower MDI scores, as shown by the lower MDI scores in infants from families in the lower SES group (Table 6).

These results are consistent with previous studies showing that SES variables likely influence early cognitive development, especially when the variables measured are maternal education (49) and household income (43). In our investigation (as shown in Table 6), we found that the infants with less educated mothers were 1.5 times more likely to have low MDI scores than the infants with more educated mothers. In addition, the infants in families with low income were 1.42 times more likely to have low MDI scores than the infants in families with higher income.

Maternal education has been shown to be a strong correlate of children's language, cognitive, and academic development. A longitudinal database (19) from the National Institute of Child Health and Human Development Study found that maternal education is associated with concurrent improvements in school readiness, language skills, and the quality of home environments in children at age 3. Other studies where interventions supporting parent-child interactions to enhance motor control and coordination are provided weekly at home from the age of 6–12 months, the overall cognitive level, especially in verbal performance, was higher at age 4 (29, 50–53). Previous investigations on low birth weight infants showed that their behavioral characteristics are also affected by family SES, which may play a role in delayed cognitive developmental at 18–22 months (52, 53). In two studies, high doses of DHA supplementation were given at an early age and found to be beneficial for improving mental development, especially in girls (50, 51). From the results of our current study, we propose that to improve their cognitive level during the stages of infant growth, early interventions are highly essential in infants with abnormal BAEP assessments and low MDI/PDI scores.

### Limitations of the Study

One of our limitations is that this is an exploratory investigation and retrospective study, the study findings should be proved in confirmatory studies in the future. In addition to this limitation, while we randomly selected our study sample, we were limited by the selection criteria that only allowed us to choose infants with perinatal issues. For this reason, selection bias may have influenced the representativeness of our study. Also, because we followed the “Ethical Review Measures for Biomedical Research Involving Human Beings” to respect the voluntary will of the subjects and abide by the principles of benefit, non-harm, and justice, we were unable to have a control group of normal infants because our study would not benefit healthy infants by administering numerous tests and assessments. Future case-control studies may also be needed to investigate the odds radios which would be more rigorous than the analyses used in the current study, with respect to the association between independent and dependent variables.

Another limitation is the relatively small number of preterm infants with clinical issues at birth enrolled in our study to examine the BAEP and MDI/PDI scores. Further studies will be needed to include higher numbers of infants with jaundice, infection, and intracranial bleeding. We were not informed of the severity of intracranial hemorrhage of the infants in our study because they had been released from the hospital following normalization of their cranial MRIs. In our next study, we will take multiple factors into consideration, including more detailed information on the severity of intracranial hemorrhage and the other perinatal conditions.

We used the Bayley II scale in the assessments, and, in studies by others using the Bayley III scale, our data may not be comparable because the Bayley III scale might reveal different results with respect to the current data. We have been using the Bayley II scale in assessments at our hospital for many years, and our trained professionals are skilled to conduct the Bayley II assessments. A comparison study between the Bayley II scale and he Bayley III scale assessments might be interesting to conduct in this area of research.

In addition, issues related to environmental factors and parental lifestyle factors such as domestic smoking, alcohol use, and nutritional factors may need to be taken into consideration in future studies of cognitive and motor neurodevelopment in infants and children. Future large scale, long-term studies are needed to provide further information concerning neurodevelopmental outcomes from birth to childhood.

## Conclusion

Our study found that BAEP values outside the normal range at age 6 months have a predictive effect on developmental delays in cognitive and motor skills, as shown by MDI scores, especially in premature newborns. Preventive prenatal care and early diagnoses and treatments during the perinatal period could possibly help prevent later problems with neurodevelopment in early childhood. For infants who experience prematurity and asphyxia, early interventions to improve cognitive and motor skills development for these infants might help to attenuate the abnormal neurodevelopmental issues that develop at later stages in these premature infants. In addition, for families with lower SES, early public health interventions, such as parental instruction with respect to teaching skills and games to enrich infant learning, may facilitate cognitive and motor development in babies with BAEP values that predict developmental delays and lower MDI scores.

## Data Availability Statement

The data analyzed during the current study are not publicly available, because they include personal identifiers and medical information that cannot be released, but are available from the first author on reasonable request.

## Ethics Statement

The studies involving human participants were reviewed and approved by Institutional Ethics Committee of Hubei Maternal and Child Health Hospital. Written informed consent to participate in this study was provided by the participants' legal guardian/next of kin.

## Author Contributions

XW designed the study, performed the experiments, and helped with manuscript preparation. XC revised the subsequent drafts of the manuscript, helped with statistical analysis, and was responsible for the final submission. HW provided general support for the research project. PZ collected and analyzed the data. JS wrote the first draft of the manuscript. SL-W reviewed and edited the subsequent drafts of the manuscript. All authors read and approved the final version of the manuscript.

## Conflict of Interest

The authors declare that the research was conducted in the absence of any commercial or financial relationships that could be construed as a potential conflict of interest.
